# *In Vitro* Optimization of Enzymes Involved in Precorrin-2 Synthesis Using Response Surface Methodology

**DOI:** 10.1371/journal.pone.0151149

**Published:** 2016-03-14

**Authors:** Huan Fang, Huina Dong, Tao Cai, Ping Zheng, Haixing Li, Dawei Zhang, Jibin Sun

**Affiliations:** 1 Tianjin Institute of Industrial Biotechnology, Chinese Academy of Sciences, Tianjin, China; 2 Key Laboratory of Systems Microbial Biotechnology, Chinese Academy of Sciences, Tianjin, China; 3 University of Chinese Academy of Sciences, Beijing, China; 4 Sino-German Joint Research Institute, Nanchang University, Nanchang, Jiangxi, China; IPK, GERMANY

## Abstract

In order to maximize the production of biologically-derived chemicals, kinetic analyses are first necessary for predicting the role of enzyme components and coordinating enzymes in the same reaction system. Precorrin-2 is a key precursor of cobalamin and siroheme synthesis. In this study, we sought to optimize the concentrations of several molecules involved in precorrin-2 synthesis *in vitro*: porphobilinogen synthase (PBGS), porphobilinogen deaminase (PBGD), uroporphyrinogen III synthase (UROS), and S-adenosyl-l-methionine-dependent urogen III methyltransferase (SUMT). Response surface methodology was applied to develop a kinetic model designed to maximize precorrin-2 productivity. The optimal molar ratios of PBGS, PBGD, UROS, and SUMT were found to be approximately 1:7:7:34, respectively. Maximum precorrin-2 production was achieved at 0.1966 ± 0.0028 μM/min, agreeing with the kinetic model’s predicted value of 0.1950 μM/min. The optimal concentrations of the cofactor S-adenosyl-L-methionine (SAM) and substrate 5-aminolevulinic acid (ALA) were also determined to be 200 μM and 5 mM, respectively, in a tandem-enzyme assay. By optimizing the relative concentrations of these enzymes, we were able to minimize the effects of substrate inhibition and feedback inhibition by S-adenosylhomocysteine on SUMT and thereby increase the production of precorrin-2 by approximately five-fold. These results demonstrate the effectiveness of kinetic modeling via response surface methodology for maximizing the production of biologically-derived chemicals.

## Introduction

**Fig 1:**
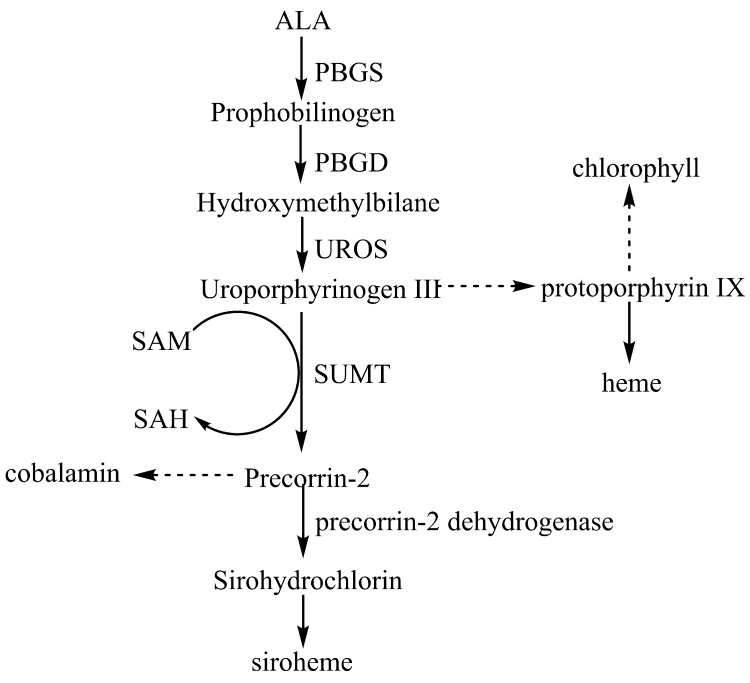
Synthesis pathway of tetrapyrroles. ALA, 5-aminolevulinic acid; PBGS, porphobilinogen synthase; PBGD, porphobilinogen deaminase; UROS, uroporphyrinogen III synthase; SUMT, S-adenosyl-l-methionine-dependent urogen III methyltransferase; SAM, S-adenosyl-l-methionine; SAH, S-adenosylhomocysteine

Tetrapyrroles such as heme, chlorophyll, siroheme, and cobalamin play essential roles in fundamental metabolic processes, including electron transfer, photosynthesis, and enzyme catalysis [1–3]. Tetrapyrroles are synthesized via a complex pathway involving multiple reactions. The first committed precursor of all tetraphyrroles is 5-aminolevulinic acid (ALA), which undergoes asymmetric condensation and deamination, a reaction catalyzed by porphobilinogen synthase (PBGS), to form porphobilinogen (PBG). Four molecules of PBG are subsequently polymerized by PBG deaminase (PBGD) to form tetrapyrrole hydroxymethylbilane (HMB) [4]. HMB is further cyclized by uroporyphyrinogen III synthase (UROS) to produce uroporphyrinogen (urogen) III. Subsequently, urogen III may be diverted into one of three pathways: heme production, chlorophyll production, or cobalamin and siroheme production (Fig 1). S-adenosyl-L-methionine (SAM)-dependent urogen III methyltransferase (SUMT) is a key enzyme in the biosynthetic pathway of cobalamin and siroheme and catalyzes the SAM-dependent bismethylation of its substrate, urogen III, to form precorrin-2 [5]. This enzyme is inhibited by urogen III and its by-product, S-adenosylhomocysteine (SAH) [6, 7]. Due to the weakness of SUMT and its competitive relationship between cobalamin and other tetrapyrrole compounds, cobalamin does not accumulate in abundance inside cells. To maximize cobalamin production, therefore, fine-tuning of the committed pathway of tetrapyrrole compounds is required.

One challenge metabolic engineers face is that of building a robust cell factory and adjusting relevant modules in order to direct flux towards a target chemical. While there are many models for predicting pathway flux, these models ignore complex regulatory interactions that result in non-linear kinetics and are often affected by slight genetic changes and strain cultivation conditions [8]. Most fine-tuning strategies focus on modular engineering or regulation of expression elements *in vivo*. A modular biosynthetic pathway for L-tyrosine production was constructed in *Escherichia coli* by expressing the enzymes necessary for converting erythrose-4-phosphate (E4P) and phosphoenolpyruvate (PEP) to L-tyrosine on two plasmids [9]. The bottlenecks in the pathway were relieved by modifications in plasmid copy numbers, promoter strength, gene codon usage, and the placement of genes in operons. The resulting strain was optimized to increase L-tyrosine yield to more than 2 g/L at 80% of the theoretical yield. In another example, in order to construct a recombinant *E*. *coli* strain that could synthesize and store high levels of triacylglycerols, changes were made to promoters, gene organization, and plasmid copy number to modulate the expression levels of the two dedicated TAG biosynthesis genes *SCO0958* and *lppβ* from *Streptomyces coelicolor* [10].

In addition to replacing expression elements, there are more complex strategies for improving chemical production, such as generating libraries of promoters [11], ribosome binding sites [12], or tunable intergenic regions (TIGRs) [13]. Optimizing gene expression using these methods, however, comes with certain disadvantages. First, the steady state of enzymes is often not taken into account. These methods are also susceptible to interference from other competitive pathways, and some of these approaches can only be used to tune one gene at a time. Lastly, the task of library building can be tedious and time-consuming. In short, these strategies are not ideal for precisely adjusting the level of enzymes in the target pathway.

Steady-state analysis of a pathway *in vitro* can minimize these disadvantages. A cell-free system was utilized in the quantitative investigation of the fatty acid biosynthesis pathway and its regulation in *E*. *coli* [14]. Fatty acid synthases were reconstituted in order to quantify the steady-state kinetic parameters and the influences of substrate, cofactor, subunit, and product concentrations [15]. Nevertheless, the enzyme components in the reaction mixture were not prepared in a coordinated manner, affecting the optimal ratio of enzymes. A more accurate method is needed to tune enzymes involved in the same pathway simultaneously. Response surface methodology (RSM) is a technique that allows for the investigation of several independent variables simultaneously to determine the optimal factorial combination of variables that results in the maximum response. RSM has been used effectively to optimize parameters in fermentation processes and other biotechnological processes [16, 17]. This method can also distinguish interaction effects from the integrated effects of individual components [18].

In this study, we aimed to relieve the bottleneck in the precorrin-2 synthesis pathway and maximize precorrin-2 productivity. A tandem-enzyme assay was carried out to produce precorrin-2 *in vitro*. Concentrations of SAM and ALA were first optimized in the reaction system. RSM was then employed to tune the rest of the relevant enzyme concentrations of the reaction system in order to motivate them to work coordinately. In the resulting optimal reaction system, precorrin-2 productivity was increased by approximately five-fold. We also observed a decrease in substrate inhibition and feedback inhibition of SUMT by SAH.

## Materials and Methods

### Chemicals and reagents

We obtained Q5 High-Fidelity DNA polymerase, restriction endonucleases, T4 DNA ligase, and Color Pre-stained Protein Standard from New England Biolabs (USA). Taq PCR Master Mix and DNA ladder were ordered from Tiangen (Beijing, China). Oligonucleotide primers were synthesized by GENEWIZ (Suzhou, China). Plasmids were extracted by E.Z.N.A TM Plasmid Miniprep Kit (Omega Bio-Tek, Inc, USA). Gel extraction and purification were performed by E.Z.N.A TM Gel Extraction Kit (Omega Bio-Tek, Inc, USA) and E.Z.N.A TM Cycle Pure Kit (Omega Bio-Tek, Inc, USA), respectively. We purchased ALA from Adamas Reagent Corporation (China), and SAM was obtained from Xiya Reagent Corporation (China).

### Plasmid construction

Genes for *hemB*, *hemC*, *hemD*, and *cobA* were individually amplified from *Pseuomonas denitrificans* genomic DNA by PCR with corresponding primers (S1 Table). *Sirc* was amplified by PCR from *Bacillus megatherium* genomic DNA using the primers SirC-F and SirC-R, including corresponding restriction sites *BamH* I and *Xho* I, respectively (S1 Table). These genes were digested with *BamH* I and *Xho* I and ligated into a pET28a (+) plasmid (Novagen), which had been digested with the same restriction endonucleases. *E*. *coli* DH5α was used as the host for cloning. *E*. *coli* BL21 (DE3) was used for gene expression.

### Protein purification

For the production of recombinant proteins, *E*. *coli* BL21 (DE3) cells carrying the recombinant plasmid were grown at 37°C in 1 L Luria–Bertani (LB) medium supplemented with kanamycin to a final concentration of 50 mg/L. When an OD600 value of 0.6–0.8 was reached, we added 0.4 mM IPTG to induce protein expression. All of the following steps were performed at 4°C or on ice. After further growth at 30°C overnight, cells were harvested by centrifugation at 5,000 g/min and re-suspended in buffer A (20 mM sodium dihydrogen phosphate, 2 M sodium chloride, 30 mM imidazole, 10 mM β-mercaptoethanol, 1% (v/v) Triton-X100, pH 7.4). The cell suspension was disrupted by a JN-3000 Plus homogenizer at 1,200 v and centrifuged at 11,000 g/min. The supernatant was filtered using a 0.22 μm filter. The filtrate was then loaded onto an equilibrated Ni^2+^-Sepharose column (GE Healthcare). After washing three times with 10 column volumes of buffer B (20 mM sodium dihydrogen phosphate, 2 M sodium chloride, 100 mM imidazole, pH 7.4), the proteins were eluted by buffer C (20 mM sodium dihydrogen phosphate, 0.5 M sodium chloride, 500 mM imidazole, pH 7.4). The protein storage buffer was then exchanged for buffer I (50 mM Tris-HCl, pH 7.5, 150 mM sodium chloride, 10% (v/v) glycerol) through ultrafiltration with Millipore’s Amicon® Ultra-15 centrifugal filter and stored at -20°C. The protein contents of the samples were analyzed by SDS-PAGE. Protein quantification was performed by 2-D quant kit (General Electric Company), using bovine serum albumin as a standard.

### Tandem-enzyme assay to produce precorrin-2 *in vitro*

Each 100 μL assay mixture contained ALA, SAM, NAD, and the five enzymes PBGS, PBGD, UROS, SUMT, and precorrin-2 dehydrogenase in buffer II (50 mM Tris-HCl, pH 8.0, 100 mM potassium chloride, 5 mM magnesium chloride, 50 mM sodium chloride, 5 mM DTT), as previously published [19]. All components were degassed beforehand. The assay mixture without ALA was pre-incubated in 96-well plates at 37°C for 10 min. The reaction was then initiated by adding ALA. Precorrin-2 was converted to sirohydrochlorin for quantification using the published extinction coefficient of sirohydrochlorin (Ɛ 376 nm = 2.4 x 10^5^ M^-1^ cm^-1^) [20]. The initial velocity of the reaction was measured from 3 to 20 minutes after initiation.

### Spectroscopy analysis

The UV-visible absorption spectra (300–700 nm) of the reaction products were recorded on a Spectra Max M5 (Molecular Devices, USA) to monitor the reaction process. Kinetic tests were also performed on a Spectra Max M5 to determine the initial velocity of the reaction. Absorption at 376 nm (the absorption of sirohydrochlorin) was recorded every 30 s.

### Response surface methodology experiment

Four-factor Box-Behnken design (BBD) was employed to investigate the response of four independent variables, representing the concentrations of PBGS, PBGD, UROS, and SUMT. Experimental design, model calculation, graph drawing, and other analyses were performed using Design Expert software (Version 8.0.6, Stat-Ease Inc., Minneapolis, USA). A quadratic polynomial model was applied to evaluate the response of the dependent variables:

$$Y_{i}=\beta_{0}+\sum_{i=1}^{4}\beta_{i}X_{i}+\sum_{i=1}^{4}\beta_{i}{}_{i}X_{i}{}^{2}+\sum_{i=1}^{3}\sum_{j=2}^{4}\beta_{ij}X_{i}X_{j}$$ (1)

where Yi is the response value, X_i_ are the coded values of the factors, β_0_ is a constant coefficient, β_i_ are the linear coefficients, β_ii_ are the quadratic coefficients, and β_ij_ are the interaction coefficients [21–23]. In this study, X_1_, X_2_, X_3_, and X_4_, correspond to the concentrations of PBGS, PBGD, UROS, and SUMT, respectively.

A total of 29 experiments were conducted. The response surface model was assessed using analysis of variance (ANOVA) to determine significance and adequacy of the model.

## Results

### Expression and purification of enzymes involved in precorrin-2 synthesis

Native PBGS, PBGD, UROS, SUMT, and precorrin-2 dehydrogenase from *Sinorhizobium meliloti* or *Bacillus megatherium* fused to an N-terminal His-tag were produced in recombinant *E*. *coli* after incubation at 30°C overnight. Purified proteins were analyzed by SDS-PAGE (S1 Fig). The molecular weights of the enzymes ranged from 25 to 36 kDa.

### Establishment of a multiple enzyme system to produce precorrin-2

**Fig 2:**
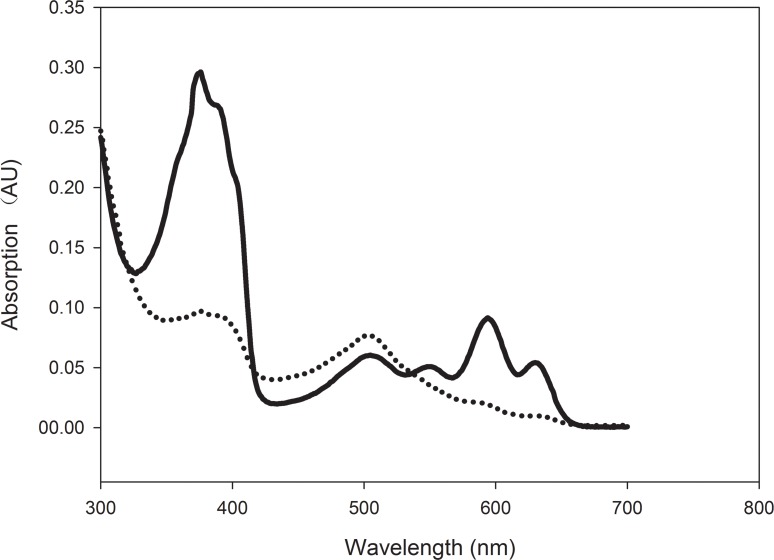
UV-visible absorption spectra of products in the tandem enzyme assay. Precorrin-2 was produced from ALA by the tandem-enzyme reaction system containing purified, recombinant PBGS, PBGD, UROS, SUMT, and SAM (dotted line); precorrin-2 was then converted into sirohydrochlorin by precorrin-2 dehydrogenase in the presence of NAD (solid line).

Initially, ALA, NAD, and all enzyme concentrations were set at 1 μM. To verify if precorrin-2 was successfully produced in the reaction mixture, we conducted an assay using precorrin-2 dehydrogenase. Sirohydrochlorin, produced from precorrin-2 by precorrin-2 dehydrogenase, has a known absorption peak of 376 nm [24, 25]. When precorrin-2 dehydrogenase was added to the reaction mixture, an absorption peak at 376 nm appeared, indicating that precorrin-2 had been transformed into sirohydrochlorin (Fig 2). To ensure that all of the precorrin-2 had been converted to sirohydrochlorin, we explored the proper precorrin-2 dehydrogenase concentration for the reaction. Varying concentrations of precorrin-2 dehydrogenase were added to the reaction mixture. The initial velocity of the reaction did not differ with precorrin-2 dehydrogenase concentrations from 0.5 μM to 10 μM. We therefore chose to use 1 μM precorrin-2 dehydrogenase for further experiments.

### Optimization of SAM cofactor concentration

**Fig 3:**
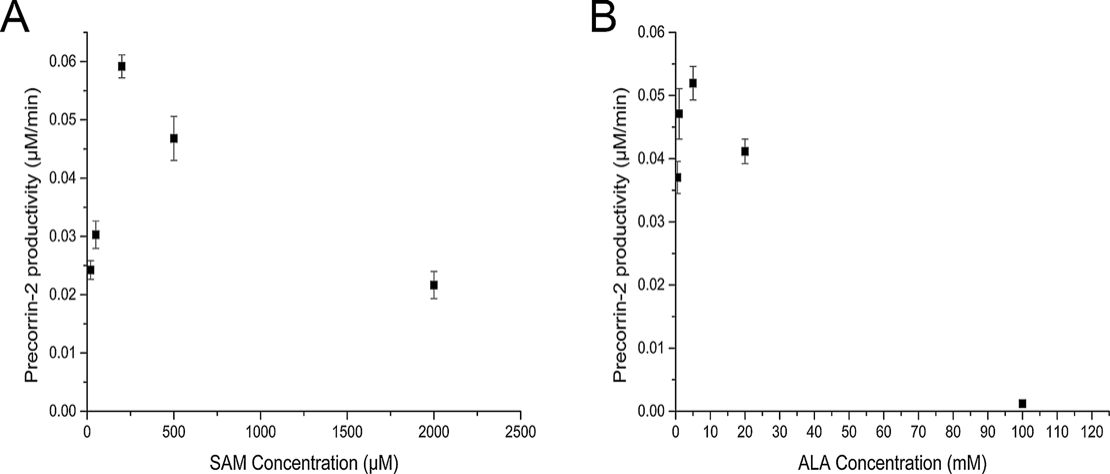
Titration of substrates and cofactors. (A), Optimization of SAM concentration. The reaction mixture contained: 1 mM ALA, 200 μM NAD, 1 μM each enzyme, and various concentrations of SAM (20 μM, 50 μM, 200 μM, 500 μM, and 2 mM SAM); (B), Optimization of ALA concentration. The reaction mixture contained: 200 μM SAM, 200 μM NAD, 1 μM each enzyme, and various concentrations of ALA (0.5 mM, 1 mM, 5 mM, 20 mM, and 100 mM). Results are presented as mean ± SD. Error bars represent standard deviations of three biological replicates.

SAM is the cofactor for precorrin-2 synthesis. SUMT is sensitive to inhibition by SAH and demonstrates a competitive relationship with SAM [26]. In order to determine the optimal SAM concentration for facilitating a forward reaction, SAM was titrated into the reaction mixture. In the initial experiment, ALA, NAD, and all enzyme concentrations were set at 1 μM. For determining the optimal concentration of SAM, we carried out the reaction using 20 μM, 50 μM, 200 μM, 500 μM, and 2 mM SAM. As SAM concentration increased from 20 μM to 200 μM, precorrin-2 productivity rose sharply. As SAM concentration rose above 200 μM, however, precorrin-2 productivity began to decline gradually (Fig 3A).

### Optimization of ALA and NAD concentrations

SUMT is inhibited at uroporphyrinogen III concentrations above 2 μM [26]. As the concentration of ALA has a direct effect on uroporphyrinogen III concentration, we sought the optimal ALA concentration. For this experiment, all enzyme concentrations were 1 μM, SAM concentration was 200 μM, and NAD concentration was 200 μM. The concentrations of ALA tested were 0.5 mM, 1 mM, 5 mM, 20 mM, and 100 mM. Similar to the pattern observed with varying SAM concentrations, precorrin-2 productivity rose with increasing ALA initially and peaked when the ALA concentration reached 5 mM. Precorrin-2 productivity then declined as ALA concentration rose further. Notably, when ALA concentration arrived at 100 mM, no precorrin-2 was detected. Additionally, we determined that precorrin-2 productivity at a NAD concentration of 1 μM exceeded productivity at 200 μM NAD (with all other component concentrations kept constant). Thus, NAD concentration was fixed at 1 μM in subsequent assays.

### Optimization of enzyme concentrations

**Fig 4:**
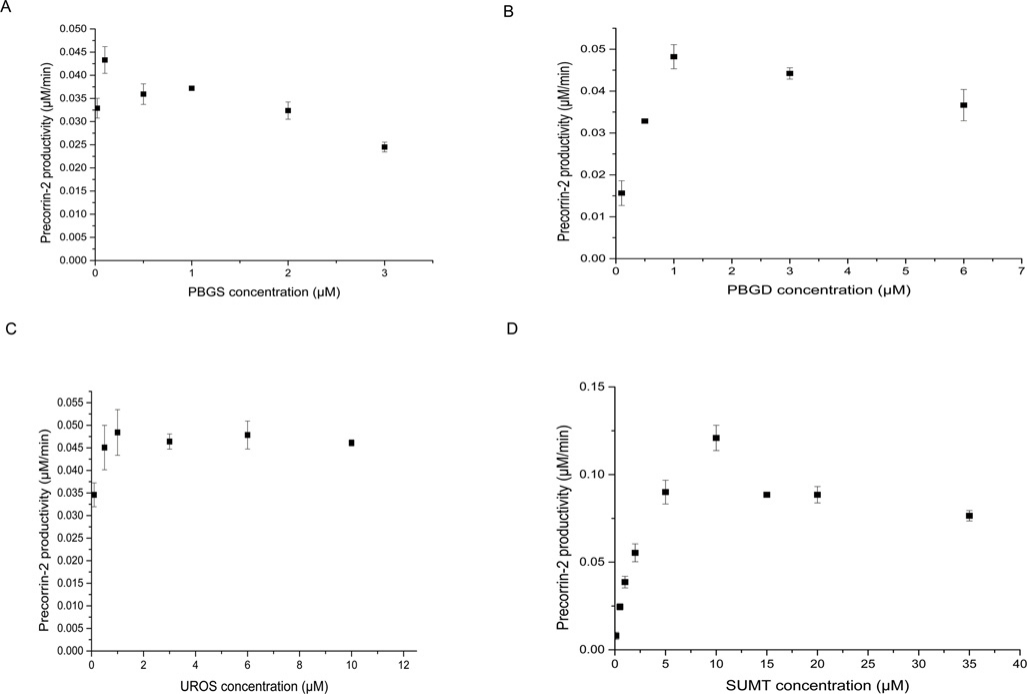
Titration of each enzyme involved in precorrin-2 synthesis. The reaction mixture contained: 5 mM ALA, 200 μM SAM, 1 mM NAD, 1 μM precorrin-2 dehydrogenase, and various concentrations of titrated enzymes. (A), Optimization of PBGS concentration. The reaction mixture contained 1 μM PBGD, 1 μM UROS, 1 μM SUMT, and various concentrations of PBGS from 0.02–3 μM. (B), Optimization of PBGD concentration. The reaction mixture contained 0.1 μM PBGS, 1 μM UROS, 1 μM SUMT, and various concentrations of PBGD from 0.1–6 μM. (C), Optimization of UROS concentration. The reaction mixture contained 0.1 μM PBGS, 1 μM PBGD, 1 μM SUMT, and various concentrations of UROS from 0.1–10 μM. (D), Optimization of SUMT concentration. The reaction mixture contained 0.1 μM PBGS, 1 μM PBGD, 1 μM UROS, and various concentrations of SUMT from 0.1–35 μM. Results are presented as the mean of 3 replicates. Error bars indicate SD.

In order to determine the optimal enzyme concentrations for each of the four enzymes, we titrated each enzyme across a range of concentrations, one enzyme at a time, in order of their function in the pathway. Specifically, PBGS, PBGD, UROS, and SUMT were tested at concentrations 0.02–3 μM, 0.1–6 μM, 0.1–10 μM and 0.1–35 μM, respectively. SAM and ALA concentrations were fixed at 200 μM and 5 mM, respectively. For the first enzyme, PBGS, all other enzyme concentrations were fixed at 1 μM. After each enzyme’s optimal concentration was determined, however, the optimal value was used in the reaction mixture when optimizing subsequent enzymes. All enzymes showed a similar bell curve pattern (Fig 4). The optimum concentrations were found to be: 0.1 μM PBGS, 1 μM PBGD, 1 μM UROS, and 10 μM SUMT.

### Model fitting and statistical analyses

Though the above experiment determined each enzyme’s optimal concentration when all other reaction ingredients were kept constant, being able to only vary the concentration of one enzyme at a time limits our ability to truly optimize precorrin-2 productivity. We therefore applied response surface methodology (RSM) to attempt to optimize the four independent variables further. Using the above preliminary experiment to determine optimal concentration ranges, experimental designs with four independent variables were created (Table 1). Each variable was assessed at three levels: -1 (the concentration immediately preceding the optimal value in the previous experiment), +1 (the concentration immediately following the optimal value in the previous experiment), and 0 (the average of the -1 and +1 concentrations). The observed and predicted initial velocities were also determined for each run of the model. The highest precorrin-2 productivity was Run 23, with all variables at the 0 level. These concentrations are very similar to the individual optimal concentrations determined in the previous experiment.

**Table 1:** Box-Behnken design (BBD) with the experimental responses.

Run	X_1_	X_2_	X_3_	X_4_	O	P
1	0.2600	1.7500	1.7500	10.0000	0.1724	0.1806
2	0.2600	1.7500	1.7500	10.0000	0.1814	0.1806
3	0.0200	3.0000	1.7500	10.0000	0.0768	0.0928
4	0.5000	1.7500	3.0000	10.0000	0.1353	0.1465
5	0.2600	0.5000	0.5000	10.0000	0.0556	0.0586
6	0.0200	1.7500	0.5000	10.0000	0.0656	0.0689
7	0.5000	1.7500	1.7500	15.0000	0.1145	0.1329
8	0.2600	0.5000	1.7500	5.0000	0.0469	0.0518
9	0.2600	1.7500	3.0000	15.0000	0.1804	0.1503
10	0.2600	1.7500	0.5000	15.0000	0.1429	0.1268
11	0.0200	1.7500	3.0000	10.0000	0.0798	0.0925
12	0.2600	1.7500	0.5000	5.0000	0.1135	0.11
13	0.2600	0.5000	3.0000	10.0000	0.056	0.0821
14	0.5000	3.0000	1.7500	10.0000	0.1686	0.1468
15	0.2600	3.0000	1.7500	15.0000	0.1473	0.1506
16	0.2600	3.0000	0.5000	10.0000	0.1334	0.1407
17	0.5000	1.7500	0.5000	10.0000	0.1172	0.123
18	0.2600	1.7500	3.0000	5.0000	0.1379	0.1335
19	0.2600	3.0000	1.7500	5.0000	0.1232	0.1338
20	0.2600	1.7500	1.7500	10.0000	0.1881	0.1806
21	0.0200	0.5000	1.7500	10.0000	0.0438	0.0107
22	0.2600	3.0000	3.0000	10.0000	0.1796	0.1642
23	0.2600	1.7500	1.7500	10.0000	0.1949	0.1806
24	0.0200	1.7500	1.7500	5.0000	0.0713	0.0621
25	0.2600	0.5000	1.7500	15.0000	0.0542	0.0685
26	0.2600	1.7500	1.7500	10.0000	0.1664	0.1806
27	0.0200	1.7500	1.7500	15.0000	0.0685	0.0789
28	0.5000	0.5000	1.7500	10.0000	0.0801	0.0648
29	0.5000	1.7500	1.7500	5.0000	0.1145	0.1162

X_1_, PBGS concentrations (μM); X_2_, PBGD concentrations (μM); X_3_, UROS concentrations (μM); X_4_, SUMT concentrations (μM); O, actual precorrin-2 productivity (μM/min); P, predicted precorrin-2 productivity (μM/min).

**Fig 5:**
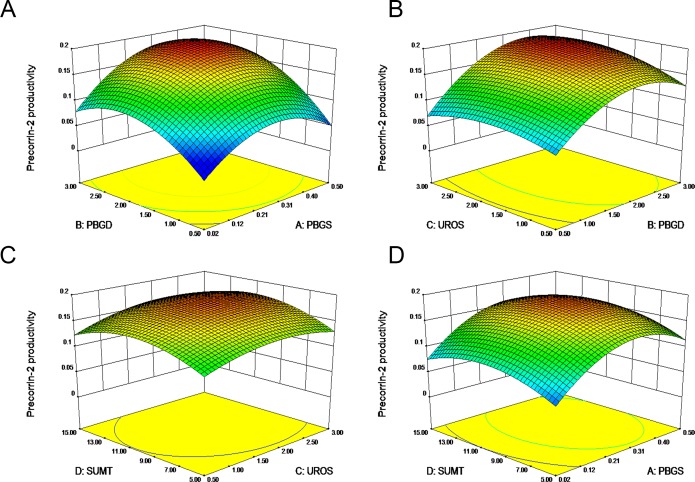
Surface response plots showing the effects of varying PBGS, PBGD, UROS, and SUMT concentrations. (A), Effect of PBGS and PBGD concentrations. (B), Effect of PBGD and UROS concentrations. (C), Effect of UROS and SUMT concentrations. (D), Effect of PBGS and SUMT concentrations.

According to the sequential sum of squares, the quadratic model was the best fit among the linear, 2FI, quadratic, and cubic models (P<0.0001). Since this model was also determined to be the best fit according to lack of fit tests (P = 0.1474) and R^2^ summary statistics (adjusted R^2^ = 0.8638, predicted R^2^ = 0.6410), we chose to apply the quadratic model for further data analyses. We assessed the significance of our RSM model by conducting an analysis of variance (ANOVA) for all independent variables and interactions (Table 2). We found all linear and quadratic effects of PBGS, PBGD, UROS, and the quadratic effect of SUMT to be significant (*P*<0.05). None of the interaction effects between these enzymes were significant. We also studied the interaction between precorrin-2 productivity and various combinations of different enzyme concentrations in which two variables were kept constant at the 0 concentration level while the other two variables were varied across their experimental ranges. These interactions were depicted using three-dimensional response surface plots (Fig 5). For example, when PBGS concentrations fluctuate from 0.02 μM to 0.5 μM, precorrin-2 productivity first increases and then declines (Fig 5A). PBGD has a similarly strong effect on precorrin-2 productivity. UROS and SUMT, however, have less of an effect on precorrin-2 productivity (Fig 5B–5D), consistent with the ANOVA analysis (Table 2).

**Table 2:** Analysis of variance (ANOVA) for response surface quadratic model.

Source	Sum of Squares	df	Mean Square	F Value	*P*-value
Model	0.0626	14	0.0045	13.6868	< 0.0001
A-PBGS	0.0089	1	0.0088	26.8329	0.0001
B-PBGD	0.0202	1	0.0202	61.7842	< 0.0001
C-UROS	0.0017	1	0.0017	5.0692	0.0409
D-SUMT	0.0008	1	0.0008	2.5822	0.1304
AB	0.0008	1	0.0008	2.3594	0.1468
AC	3.8377E-06	1	3.8400E-06	0.0117	0.9153
AD	1.9628E-06	1	1.9600E-06	0.0060	0.9393
BC	0.0005	1	0.0005	1.6036	0.2261
BD	7.0510E-05	1	7.0500E-05	0.2157	0.6495
CD	4.3106E-05	1	4.3100E-05	0.1319	0.7219
A^2^	0.0181	1	0.0181	55.2219	< 0.0001
B^2^	0.01563	1	0.0156	47.8124	< 0.0001
C^2^	0.0026	1	0.0026	8.0455	0.0132
D^2^	0.0060	1	0.0060	18.3016	0.0008
Residual	0.0046	14	0.0003		
Lack of Fit	0.0040	10	0.0004	3.04398	0.1474

We attempted to simplify the model by removing items that were insignificant at the 95% confidence level from the final quadratic model equation, with the exception of the linear coefficient of SUMT, which was kept to maintain the model’s hierarchy. We conducted a new ANOVA for the independent variables of this simplified model (Table 3). The strongest effect is seen with PBGD concentration, followed by PBGS, UROS, and SUMT concentrations. The model was found to be significant (F = 25.55, *P*<0.01). The adjusted R^2^ of 0.8752 is in reasonable agreement with the predicted value of 0.8049. The following regression Eq 2 represents the mathematical model for maximum precorrin-2 productivity:

$$\begin{array}{l} Y=-0.2584+0.5889{\text{X}}_{1}+0.1428{\text{X}}_{2}+0.0545{\text{X}}_{3}+0.02597{\text{X}}_{4}-0.9158{\text{X}}_{1}{}^{2}-0.0314\\ {\text{X}}_{2}{}^{2}-0.0129{\text{X}}_{3}{}^{2}-1.2148\text{E}-003{\text{X}}_{4}{}^{2} \end{array}$$ (2)

**Table 3:** Analysis of variance (ANOVA) for reduced response surface quadratic model.

Source	Sum of Squares	df	Mean Square	F Value	*P*-Value
Model	0.0610	8	7.6500E-03	25.5500	< 0.0001
A-PBGS	8.7700E-03	1	8.7700E-03	29.2800	< 0.0001
B-PBGD	0.0200	1	0.0200	67.4200	< 0.0001
C-UROS	1.6600E-03	1	1.6600E-03	5.5300	0.0290
D-SUMT	8.4400E-04	1	8.4400E-04	2.8200	0.1088
A^2^	0.0180	1	0.0180	60.2600	< 0.0001
B^2^	0.0160	1	0.0160	52.1700	< 0.0001
C^2^	2.6300E-03	1	2.6300E-03	8.7800	0.0077
D^2^	5.9800E-03	1	5.9800E-03	19.9700	0.0002
Residual	5.9900E-03	20	3.0000E-04		
Lack of Fit	5.4600E-03	16	3.4100E-04	2.5700	0.1869

### Experimental validation of the model

In order to verify the model’s accuracy, the actual maximum initial velocity of the reaction using all optimal concentrations was compared with the predicted value. The following optimal concentrations of the enzymes were calculated to maximize the initial velocity: PBGS at 0.32 μM, PBGD at 2.27 μM, UROS at 2.12 μM, and SUMT at 10.69 μM. This means that the optimal molar ratios are approximately 1:7:7:34. These optimal ratios reflect the fact that SUMT activity is lower than that of the other enzymes. The model’s predicted initial velocity based on these optimal concentrations is 0.195 μM/min. We performed six replicate experiments with all enzymes at their optimal concentrations. The mean initial velocity for the replicates was found to be 0.1966±0.0028 μM/min. The small difference between actual and predicted values reflects the model’s accuracy.

## Discussion

Complex chemicals are usually produced by combinations of enzymes in microorganisms. Due to toxicity of intermediates or bottlenecks in biosynthetic pathways, chemical production can be low, especially for heterologous hosts. To maximize target chemical synthesis, therefore, it is important to optimize expression of the enzymes involved [27, 28]. Precorrin-2 is a committed precursor of the cobalamin and siroheme synthesis pathway. SUMT is a key enzyme involved in cobalamin synthesis. This enzyme is inhibited by excess substrate and the product SAH. The straightforward way to relieve inhibition is to engineer an improved enzyme through directed evolution or rational design. For this, however, an efficient screening technique is needed for high throughput screening of multiple mutants. To our knowledge, there has been no reported successful engineering of this enzyme.

In our study, we produced precorrin-2 *in vitro* for kinetic analysis and optimization of precorrin-2 synthesis. As the substrate and cofactor concentrations affect precorrin-2 productivity, these molecules were optimized first in a preliminary reaction system. We then titrated the individual enzymes one at a time to determine optimal concentrations. However, the titration of individual enzymes in this way may not reflect the optimal condition of the entire synthesis pathway [14, 15, 29]. Therefore, we simulated the titration of all four enzymes involved in precorrin-2 synthesis simultaneously by RSM. As these variables were determined by RSM simultaneously, rather than one at a time and were in a narrow range based on the results of initial titration of individual enzymes, the values predicted by RSM are more reliable than those determined by titration of individual enzymes. In addition, as the enzymes were optimized in the order of their function in the pathway during the initial titration of individual enzymes, the values determined by this initial titration became increasingly similar to the values predicted by RSM as we moved through the pathway. We then confirmed increased precorrin-2 productivity at these optimal concentrations of 0.1966±0.0028 μM/min, an increase of approximately 5-fold after optimization. Notably, although SUMT is the key enzyme of the cobalamin synthesis pathway, ANOVA analysis showed that the linear and quadratic effects of SUMT were lower than for the other enzymes. This implies that substrate and feedback inhibition of SUMT can be minimized, and the metabolic flux to precorrin-2 balanced, via fine-tuning of the ratios of these enzymes.

Our analysis demonstrates that RSM is a useful tool for studying metabolic flux control. RSM could be similarly applied to fine-tune the synthesis of other metabolites. Analyses of such pathways *in vitro* can serve as references for genetic manipulations to maximize metabolite productivity. A similar method has been successfully applied to increase the production of fatty acids, fatty acid short-chain esters, fatty alcohols, farnesenes, alkenes, and alkanes [15, 29–32]. Such a method could even be used to study the production of other molecules produced *in vitro*, such as hydrogen, a prospect that may have even more significant implications for biotechnological research.

## Supporting Information

S1 Fig SDS-PAGE analysis of the production and purification of recombinant PBGS, PBGD, UROS, SUMT, and precorrin-2 dehydrogenase. Lane 1, PBGS; lane 2, PBGD; lane 3, UROS; lane 4, SUMT; lane 5, precorrin-2 dehydrogenase.
(DOCX)

S1 Table Primers used in this study. (DOCX)
