# Supplementary material for: In Vitro Optimization of Enzymes Involved in Precorrin-2 Synthesis Using Response Surface Methodology
Source: PLoS One. 2016 Mar 14;11(3):e0151149. doi: 10.1371/journal.pone.0151149 (PMC4790935; doi:10.1371/journal.pone.0151149)

**S1 Fig. SDS-PAGE analysis of the production and purification of recombinant PBGS, PBGD, UROS, SUMT, and precorrin-2 dehydrogenase.** Lane 1, PBGS; lane 2, PBGD; lane 3, UROS; lane 4, SUMT; lane 5, precorrin-2 dehydrogenase.


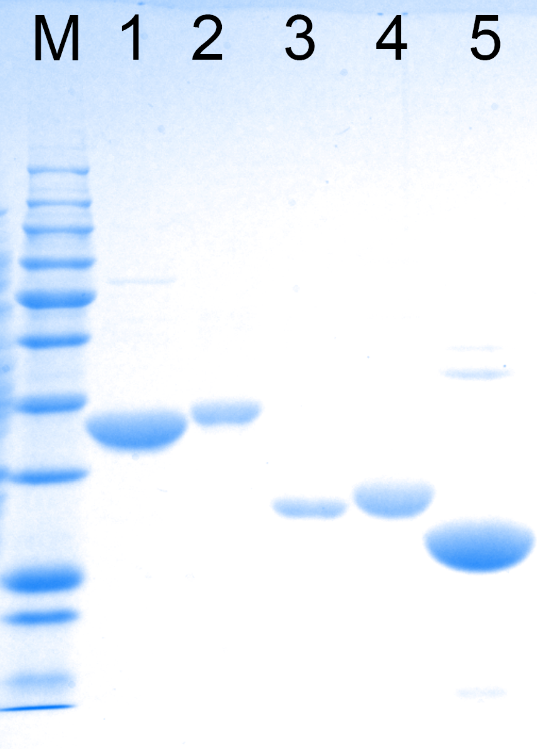

Supplement: S1 Fig — Lane 1, PBGS; lane 2, PBGD; lane 3, UROS; lane 4, SUMT; lane 5, precorrin-2 dehydrogenase. (DOCX) [file pone.0151149.s001.docx]
