# Supplementary material for: In Vitro Optimization of Enzymes Involved in Precorrin-2 Synthesis Using Response Surface Methodology
Source: PLoS One. 2016 Mar 14;11(3):e0151149. doi: 10.1371/journal.pone.0151149 (PMC4790935; doi:10.1371/journal.pone.0151149)
Supplement: S1 Table — (DOCX) [file pone.0151149.s002.docx]

**S1 Table. Primers used in this study**

| gene | primers | sequences |
| --- | --- | --- |
| *hemB* | hemB-F（*Bam*H I） | CGGGATCCGTGGACAGGGTCACCGGCCATC |
|  | hemB-R（*Xho* I） | CCGCTCGAGTCAGCGGCCTTTGGCGAGAATG |
| *hemC* | hemC-F（*Bam*H I） | CGGGATCCATGCAAACAAAACCTTTCCG |
|  | hemC-R（*Xho* I） | CCGCTCGAGTTAAGTCCAGCTTGCAAAG |
| *hemD* | hemD-F（*Bam*H I） | CGGGATCCATGCGCGTGCTCGTCACC |
|  | hemD-R（*Xho* I） | CCGCTCGAGTTAGAGAAGACTGAAAAGGC |
| *cobA* | cobA-F（*Bam*H I） | CGGGATCCATGATCGACGACCTCTTTGC |
|  | cobA-R（*Xho* I） | CCGCTCGAGTCATGCCGGGTTCCTGAG |
| *SirC* | SirC-F（*Bam*H I） | CGGGATCCATGTATACCGTTATGCTTG |
|  | SirC-R（*Xho* I） | CCGCTCGAGTTATTTATATTTTTCAATCTGTTG |
